# Hypothyroidism Causing Pericardial Effusion: A Case Report

**DOI:** 10.7759/cureus.6393

**Published:** 2019-12-16

**Authors:** Sabawoon Mirwais, Syed Hassan Kazmi, Syed I Hussain, Maiwand Mirwais, Ajay Sharma

**Affiliations:** 1 Cardiovascular Medicine, Beth Israel Deaconess Medical Center, Boston, USA; 2 Internal Medicine, University of Pittsburgh Medical Center Mercy, Pittsburgh, USA; 3 Cardiology, Lahey Hospital and Medical Center, Burlington, USA

**Keywords:** hypothyroidism, pericardial effusion

## Abstract

Hypothyroidism is an endocrine disorder with worldwide prevalence that can affect multiple organ systems. It can be asymptomatic and subclinical or overtly symptomatic with a potential to get complicated by fatal pathologies. It is an established cause of pericardial effusion, which in turn can be complicated by cardiac tamponade and severe hemodynamic instability. Herein we report the case of a 68-year-old male with history of Graves’ disease treated with radioiodine ablation and consequent hypothyroidism, presenting with moderate pericardial effusion.

## Introduction

Hypothyroidism, an endocrine disorder characterized by low or absent functional thyroid hormone, is diagnosed when decreased levels of thyroid hormones cause an increased secretion of thyroid-stimulating hormone (TSH) [1]. The clinical picture of hypothyroidism can have variable presentation, ranging from absent and subclinical symptoms to overt multiorgan failure. The clinical manifestations can vary based on the age of the patient at the time of diagnosis and the severity of hormone deficiency. The most commonly reported symptoms of hypothyroidism are weight gain, constipation, cold sensitivity, fatigue, and dry skin [2-4]. Less common signs include myopathy, carpal tunnel syndrome, and hoarseness of voice. Hypothyroidism, having the potential to affect any organ system, can be complicated by serious pathologies requiring timely intervention. One of the rare, yet serious complications of hypothyroidism is pericardial effusion [5-10]. Hypothyroidism causes increased permeability of the pericardial capillaries to albumin.This, in addition to decreased drainage of albumin into the lymphatic system, leads to increased colloid pressure within the pericardium and hence, decreased colloid osmotic pressure gradient between the pericardial space and pericardium. This culminates in the accumulation of fluid in the pericardial space [11-14]. The resultant pericardial effusion can be subclinical and can progress to an overt pathology.

## Case presentation

**Figure 1:**
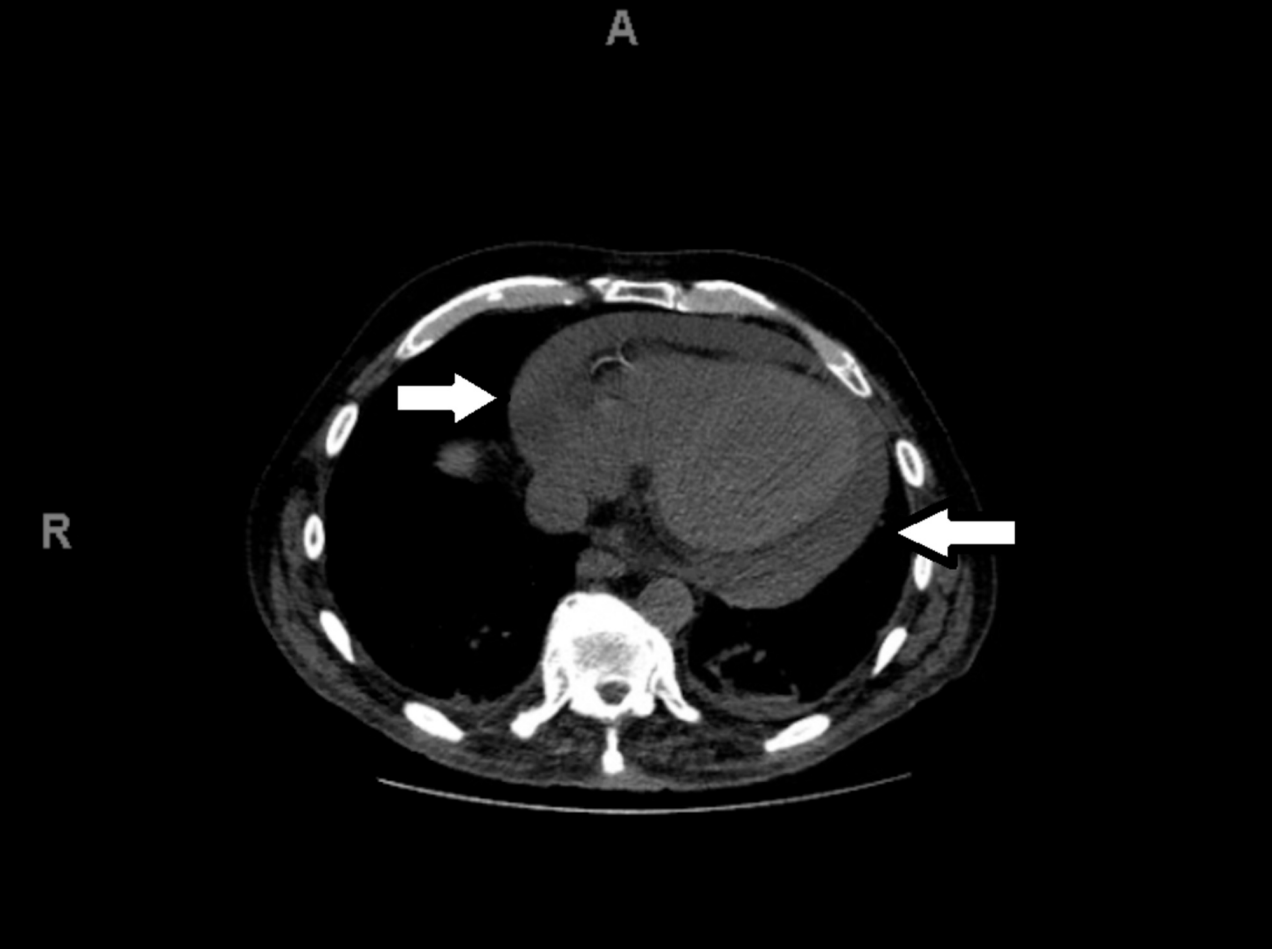
CT scan. Image demonstrating pericardial effusion (white arrows).

**Figure 2:**
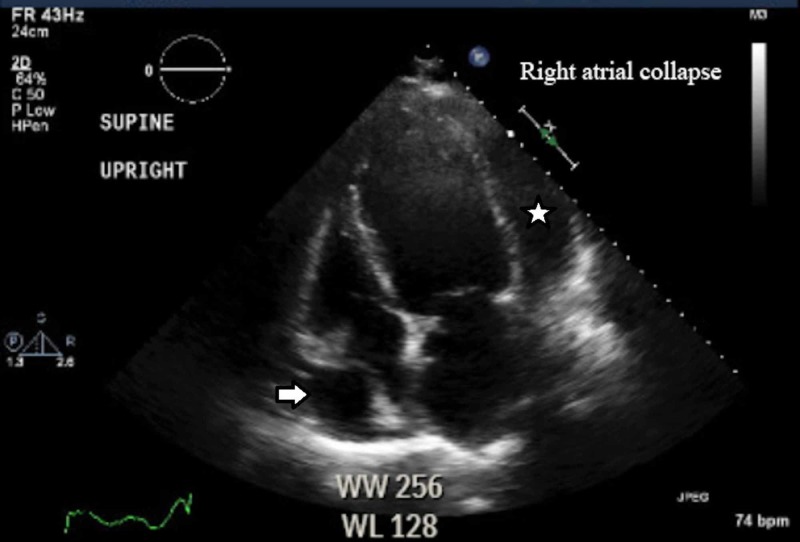
Echocardiogram. Image shows pericardial effusion (white star) and right atrial collapse (white arrow).

A 68-year-old male with an extensive medical history of alcohol and tobacco use, medication non-compliance, Graves’ disease treated with radioiodine ablation and resulting hypothyroidism, chronic obstructive pulmonary disease, hypertension, deep venous thrombosis with inferior vena cava filter placement, and bipolar disorder presented to the emergency room with alcohol intoxication and cough. The patient also reported subjective fever and chills at the time of presentation along with history of previous incarceration, shelter living, and homelessness. His initial chest x-ray showed probable pericardial effusion, which was later confirmed on chest CT scan (Figure 1) and bedside echocardiography (Figure 2). Chest CT scan also showed ground glass opacities in the bilateral lower lobes. The evaluation of patient’s presenting complaint of cough guided the way for the incidental finding of pericardial effusion on chest x-ray. Bedside ultrasound in the emergency room ruled out right ventricular collapse. Transthoracic echocardiography demonstrated early tamponade physiology, and the patient was hemodynamically stable. Based on the presentation, we suspected tuberculosis (TB) as the cause of his chronic cough. The patient was transferred to a negative pressure room for infectious evaluation. TB was ruled out after a negative purified protein derivative and QuantiFERON gold test. He was instead found to have been suffering from aspiration pneumonia after his sputum culture came back positive for Klebsiella and Serratia extended-spectrum beta-lactamase producing species. Poor dentition and usual alcohol intoxication made the patient at risk of recurrent aspiration of the oral bacteria.

 

The patient was prescribed levothyroxine 25 mcg daily for hypothyroidism in the past but he had run out of his medication several months ago. His TSH level of 31.42 μIU/mL (normal range: 0.4-5.5 μIU/mL) with undetectable free T4 and previous history of thyroid disease pointed towards uncontrolled hypothyroidism as the likely cause of his moderate pericardial effusion. The patient’s hemodynamic stability did not qualify him for the indication of pericadiocentesis. He was prescribed levothyroxine 150 mcg daily which resulted in the reduction of the TSH level to 14.17 μIU/mL in less than two weeks. The pericardial effusion was followed up with serial transthoracic echocardiogram, and the patient was discharged on the advice to follow-up with primary care physician. 

## Discussion

Our patient was suffering from multiple comorbidities at the time of presentation. His extensive medical history and unconventional presentation delayed the diagnosis of hypothyroidism as the cause of moderate pericardial effusion. Shelter living, homelessness, history of incarceration, subjective fever and chills, presenting complaint of chronic cough, and the fact that differential diagnoses of pericardial effusion include TB, led the team to suspect and provisionally diagnose pulmonary TB [15,16]. Also, with an extended medical history, our patient required urgent multidisciplinary approach. Patient’s history of previous thyroid disease and thyroid function test results established the diagnosis of hypothyroidism. Metabolic disorders, such as hypothyroidism, are considered one of the less common causes of pericardial effusion [17]. Most of the clinical symptoms of hypothyroidism can easily be reversed with thyroid hormone replacement [18]. The relatively high incidence of pericardial effusion due to hypothyroidism and the potential complications of pericardial effusion necessitate early recognition and management [6,8,9,19]. The rarity of the association between these two conditions warrants more research and investigation in order to be well prepared to provide the standard of care.

## Conclusions

Hypothyroidism with pericardial effusion is a relatively rare occurrence in clinical setting. The potential of this presentation to be complicated by life-threatening conditions, such as cardiac tamponade and hemodynamic instability, requires a high degree of clinical suspicion. Thyroxine therapy over extended period can result in alleviating mild pericardial effusion and serial echocardiography can aide in documenting the effect of treatment and change in the volume of effusion.
